# Supplementary material for: Immobilization and docking studies of Carlsberg subtilisin for application in poultry industry
Source: PLoS One. 2023 Aug 16;18(8):e0269717. doi: 10.1371/journal.pone.0269717 (PMC10431679; doi:10.1371/journal.pone.0269717)
Supplement: S1 Fig — (A) SDS-PAGE Excised Gel bands with protein marker for 80% precipitated enzyme extract (B) Excised zymogram (1% Casein) of potentially purified enzyme (C) MALDI -TOF MS/MS spectrum of a tryptic peptide with M/z 1108.5. (PDF) [file pone.0269717.s001.pdf]

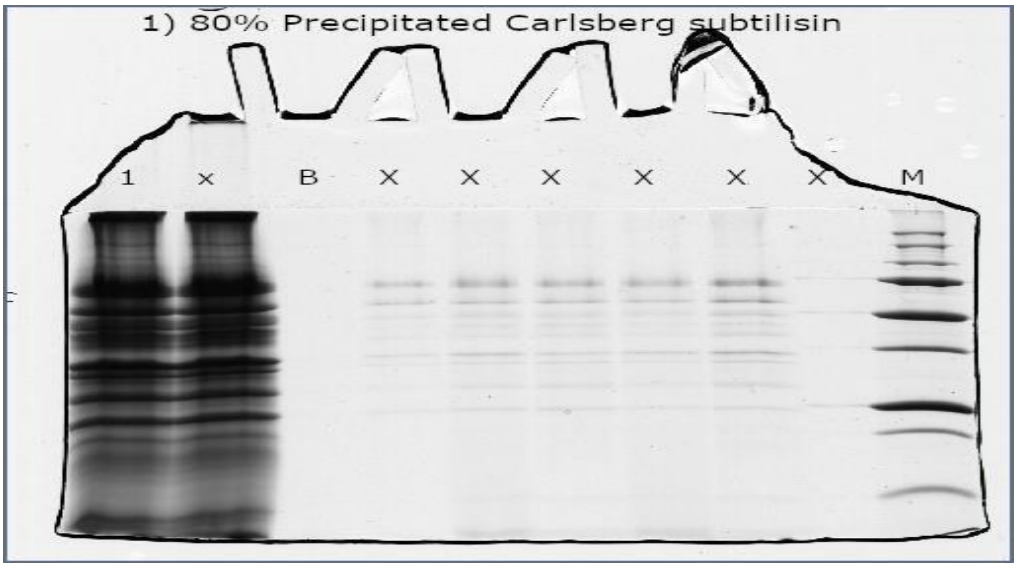

SDS-PAGE Excised Gel bands with protein marker for 80 % precipitated enzyme extract; (M) Marker; (X) Not included in the manuscript (contains the same sample in dilutions); (B) Blank; (1) 80% Precipitated Carlsberg subtilisin.

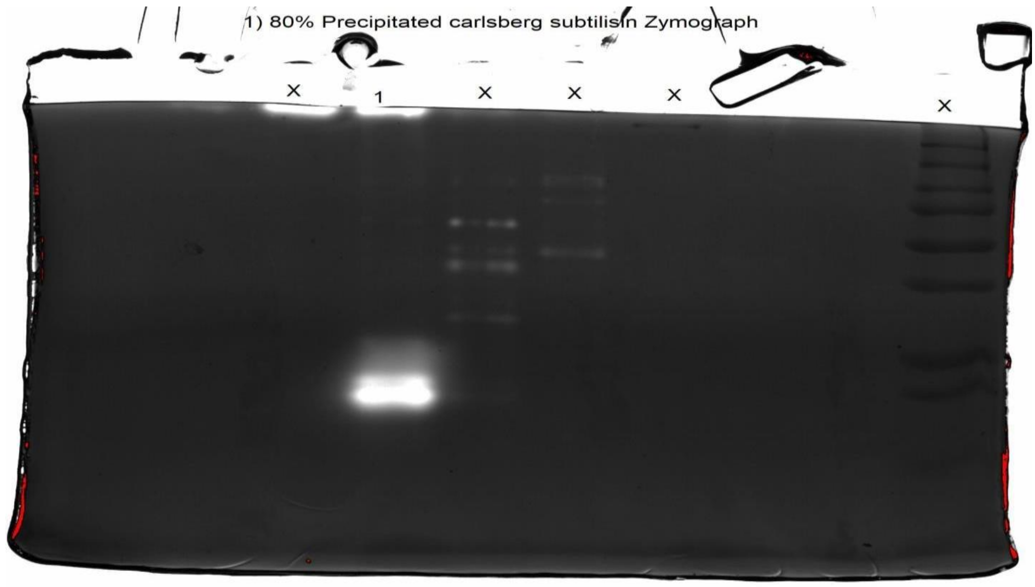

Excised zymogram (1 % Casein) of potentially purified enzyme; (X) Not included in the manuscript; (1) Active band of Carlsberg subtilisin.

S1 Figure. Carlsberg Subtilisin produced by *B. licheniformis* PB1 (A) SDS-PAGE Excised Gel bands with protein marker for 80 % precipitated enzyme extract (B) Excised zymogram (1 % Casein) of potentially purified enzyme (C) MALDI -TOF MS/MS spectrum of a tryptic peptide with M/z 1108.5
