# Supplementary material for: Immobilization and docking studies of Carlsberg subtilisin for application in poultry industry
Source: PLoS One. 2023 Aug 16;18(8):e0269717. doi: 10.1371/journal.pone.0269717 (PMC10431679; doi:10.1371/journal.pone.0269717)
Supplement: S1 Raw images — (PDF) [file pone.0269717.s002.pdf]

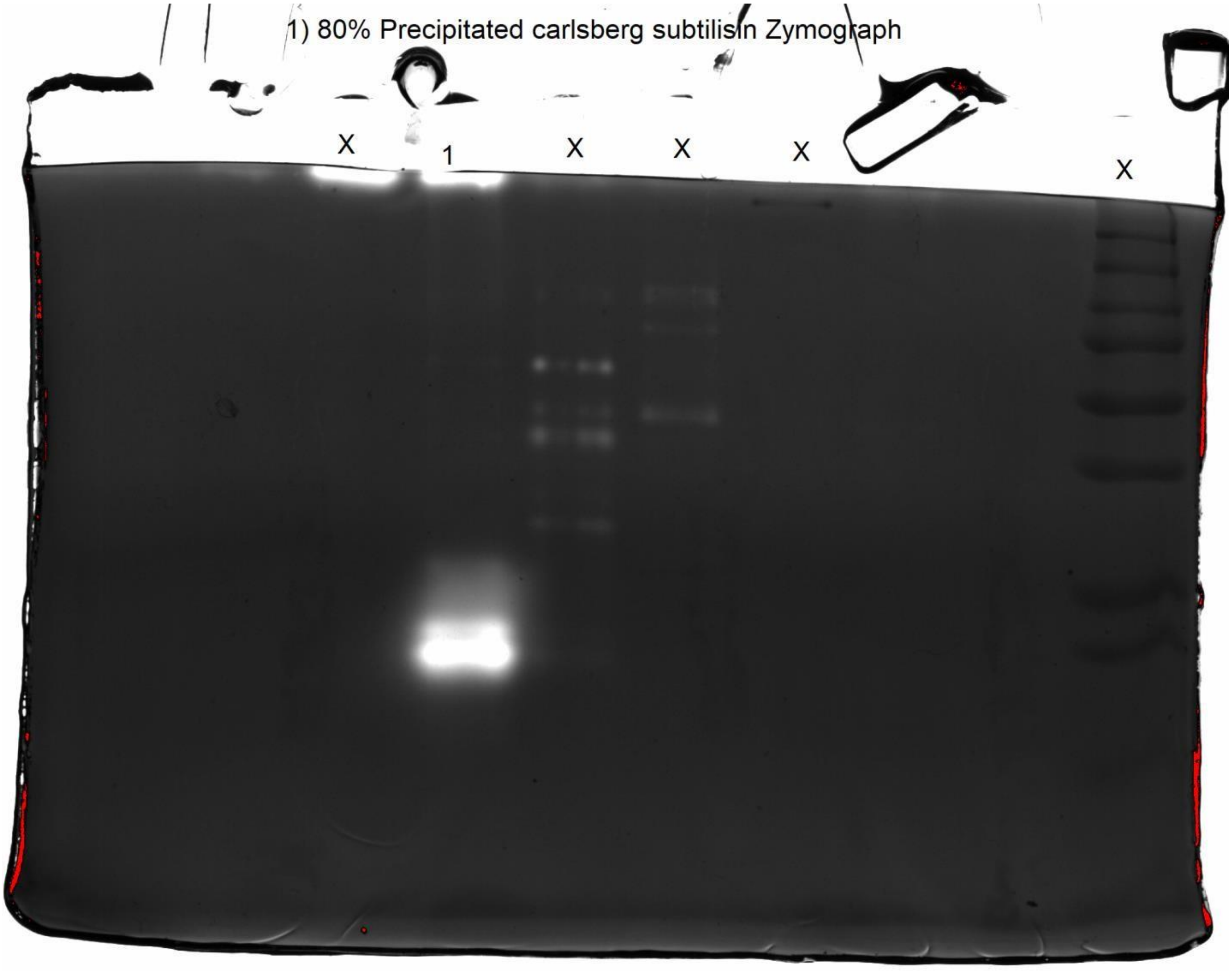

S1 Figure The Zymogram of Carlsberg subtilisin showing the activity of Carlsberg subtilisin

X) Not included in the manuscript

1) Zymogram of Carlsberg subtilisin

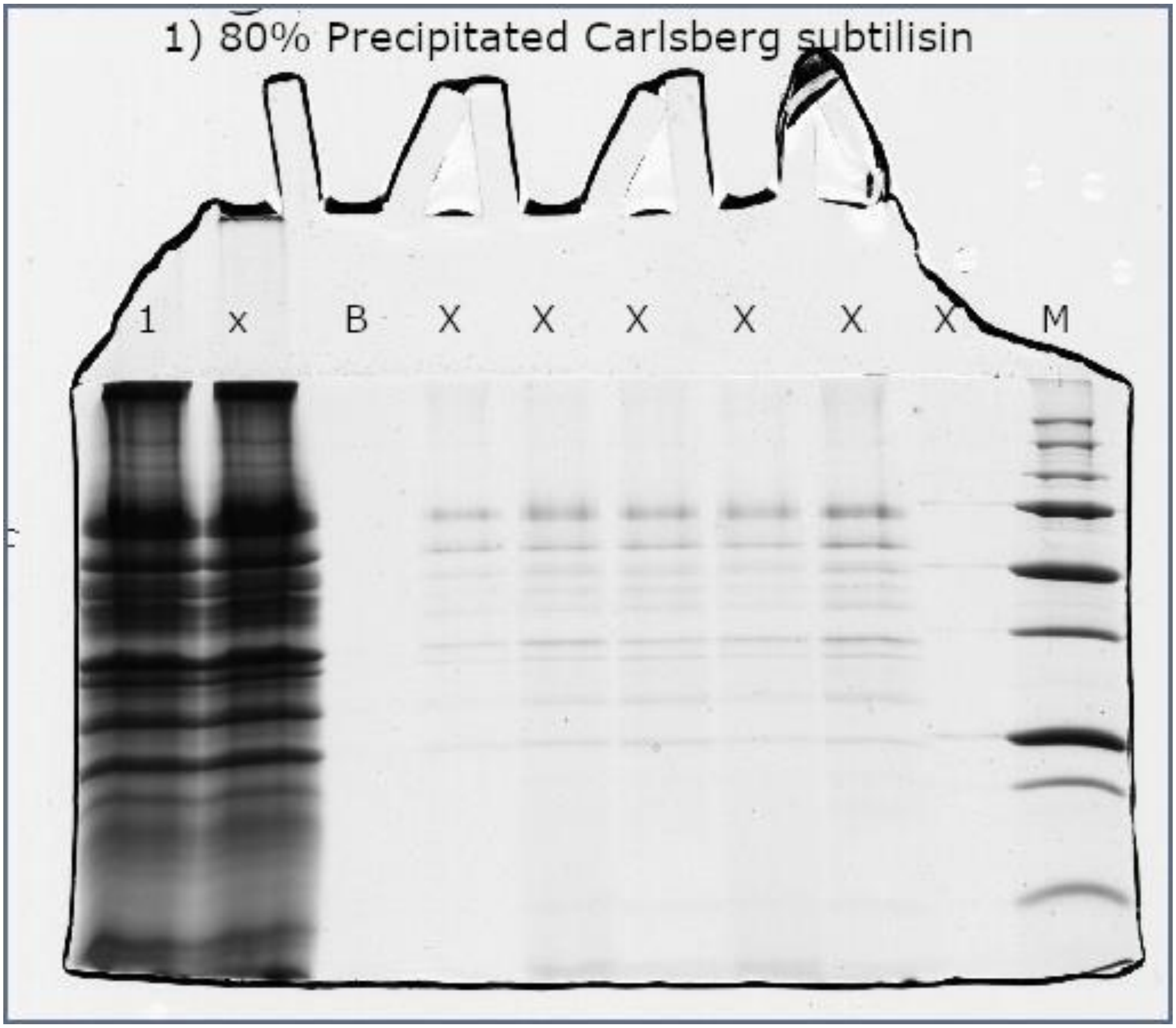

S2 Figure The SDS-PAGE of Carlsberg subtilisin and Marker.

M) Marker

X) Not included in the manuscript (contains the same sample in dilutions)

B) Blank

1) 80% Precipitated Carlsberg subtilisin
