# Supplementary material for: Immobilization and docking studies of Carlsberg subtilisin for application in poultry industry
Source: PLoS One. 2023 Aug 16;18(8):e0269717. doi: 10.1371/journal.pone.0269717 (PMC10431679; doi:10.1371/journal.pone.0269717)
Supplement: S1 File — (DOCX) [file pone.0269717.s003.docx]

**Supporting Materials**

**Fig. 1 Standard curve of BSA**

| Standard Curve of Bovine Serum Albumin | | |
| --- | --- | --- |
| Concentrations (mg/ml) | **Absorbance (650nm)** |  |
| 10 | 0.03 |  |
| 20 | 0.09 |  |
| 30 | 0.151 |  |
| 40 | 0.274 |  |
| 50 | 0.3293 |  |
| 60 | 0.365 |  |
| 70 | 0.4 |  |
| 80 | 0.4505 |  |
| 90 | 0.48455 |  |

**Table 2. Standard curve of Tyrosine**

| Concentration (mg/ml) | Tyrosine |
| --- | --- |
|  |  |
| 10 | 0.1 |
| 20 | 0.193 |
| 30 | 0.313 |
| 40 | 0.422 |
| 50 | 0.487 |
| 60 | 0.572 |
| 70 | 0.658 |
| 80 | 0.713 |
| 90 | 0.78 |
| 100 | 0.877 |

**Table 3. Enzyme Activity and total protein estimation ammonium sulphate precipitation at 50% and 80% concentrations.**

| Conc. of Ammonium sulphate |  | Enzyme Activity (U/ml/min) |  | Total protein absorbance | Total protein (mg/ml) |  |
| --- | --- | --- | --- | --- | --- | --- |
| 50 % | Supernatant | 76.02 |  | 0.579 | 100.46 |  |
|  | Pellet | 983.02 |  | 1.6885 | 291.42 |  |
| 80 % | Supernatant | 80.25 |  | 0.817 | 141.43 |  |
|  | Pellets | 1128.53 |  | 4.55 | 783.94 |  |

*Enzyme control and substrate controls were subtracted from the absorbance at the time of Absorbance reading step.

**Table 4. Enzyme activity of crude enzyme for immobilization of solid support material.**

| Carbon Source | Enzyme Activity (U/ml/min) | Total Protein (mg/ml) | Specific Activity (U/mg) |
| --- | --- | --- | --- |
| Wheat Bran | 845.27 | 46.08 | 18.34 |
| Soyabean Meal | 845.27 | 46.08 | 18.34 |
| Bentonite | 925.65 | 60.42 | 15.32 |

**Table 4. Enzyme activity of solid support selection for immobilization.**

| Solid Support | Incubation | Abs. *dil. | Enzyme Activity (U/ml/min) | Total Protein (mg/ml) | Specific Activity (U/mg) | Specific Activity  (mg/ml)  (B-A) |
| --- | --- | --- | --- | --- | --- | --- |
| Wheat Bran | Before (A) | 2.113 | 273.108 | 783.94 | 0.348 |  |
|  | After (B) | 3.559 | 464.37 | 783.94 | 0.592 | 0.244 |
| Soyabean Meal | Before (A) | 2.842 | 369.53 | 783.94 | 0.471 |  |
|  | After (B) | 4.826 | 631.97 | 783.94 | 0.806 |  |
|  |  |  |  |  |  | 0.335 |

**Table 5. Carlsberg subtilisin adsorption on inorganic support bentonite.**

| Sr. No. | Enzyme Activity  U/ml/min | Total Protein  (mg/ml) | Specific activity  (mg/ml) | Percent Adsorption (U/ml) |
| --- | --- | --- | --- | --- |
| 1 | 348.968 | 766.73 | 0.455 | 30.922 |
| 2 | 269.702 | 766.73 | 0.352 | 23.899 |

**Parameter Optimizations for Free enzyme and Immobilized Enzyme by using OVAT technique.**

**Table 6. Effect of change in buffer molarity**

| Molarity of buffer | Free enzyme  Specific Activity  (U/mg) | | Immobilized enzyme  Specific Activity  (U/mg) | |
| --- | --- | --- | --- | --- |
|  | Replicate 1 | Replicate 2 | Replicate 1 | Replicate 2 |
| 0.1 | 3 | 2.32 | 1 | 1.23 |
| 0.2 | 3.9 | 2.89 | 4.56 | 5.4 |
| 0.3 | 3.5 | 3.2 | 4.29 | 4 |
| 0.4 | 5.1 | 4.5 | 6.78 | 5 |
| 0.5 | 5.9 | 5.43 | 6.11 | 6.29 |
| 0.6 | 10.6 | 9.87 | 11.23 | 10.98 |
| 0.7 | 10.8 | 9.43 | 10.23 | 10.43 |
| 0.8 | 8.9 | 7.91 | 11 | 10.93 |
| 0.9 | 11.3 | 10.21 | 14.45 | 16.23 |
| 1 | 14.4 | 15.21 | 16.32 | 17.08 |
| 1.1 | 5.3 | 5.65 | 13.43 | 14.23 |
| 1.2 | 9.7 | 9.01 | 10.23 | 11.2 |

**Table 7. Effect of pH change on free and immobilized Carlsberg subtilisin.**

| Buffer pH | Free enzyme  Specific Activity  (U/mg) | | Immobilized enzyme  Specific Activity  (U/mg) | |
| --- | --- | --- | --- | --- |
|  | Replicate 1 | Replicate 2 | Replicate 1 | Replicate 2 |
| 2 | 4.32 | 4.81 | 3.45 | 2.5 |
| 3 | 5.88 | 3.43 | 4.4 | 3.2 |
| 4 | 3.28 | 8.04 | 8.9 | 6.7 |
| 5 | 4.05 | 4.07 | 10.23 | 11.2 |
| 6 | 7.94 | 6.87 | 14.45 | 12.21 |
| 7 | 16.67 | 8.12 | 21.25 | 20.43 |
| 8 | 30.01 | 30.3 | 36.32 | 34.2 |
| 9 | 12.25 | 11.3 | 26.23 | 21.45 |

**Table 8. Effect of change in Temperature on free and immobilized Carlsberg subtilisin.**

| Temperature (C) | Free enzyme  (U/mg) | | Immobilized enzyme  (U/mg) | |
| --- | --- | --- | --- | --- |
|  | Experiment 1 | Experiment 2 | Experiment 1 | Experiment 2 |
| 20 | 11.659 | 10.21 | 10.12 | 7.9 |
| 30 | 12.496 | 13.532 | 9.9 | 12.2 |
| 40 | 12.286 | 10.87 | 16.78 | 18.75 |
| 50 | 33.006 | 34.329 | 43.5 | 49.4 |
| 60 | 24.76 | 22.51 | 32.2 | 35.54 |
| 70 | 15.752 | 11.213 | 18.45 | 20.14 |

**Table 9. Desorption of immobilized Carlsberg subtilisin.**

| Wash no. | Specific activity  (U/mg) | |
| --- | --- | --- |
|  | Replicate 1 | Replicate 2 |
| 1 | 1.6 | 1.01 |
| 2 | 2.4 | 2.12 |
| 3 | 4 | 4.24 |
| 4 | 3.3 | 3.43 |

**Table 10. Supplementation of immobilized Carlsberg subtilisin-bentonite product in poultry feed.**

|  | Body weight of birds (gm) | | | | | | |
| --- | --- | --- | --- | --- | --- | --- | --- |
|  | **Day 4** | **Day 10** | **Day 16** | **Day 22** | **Day 28** | **Day 40** | **Day 46** |
| Group 1 Protease | 49 | 175 | 351 | 524 | 778 | 1596 | 2000 |
|  | 56 | 168 | 308 | 516 | 899 | 1884 | 1700 |
|  | 47 | 149 | 392 | 563 | 978 | 1693 | 1826 |
|  | 47 | 156 | 349 | 495 | 856 | 1440 | 1755 |
|  | 48 | 161 | 332 | 570 | 825 | 1631 | 1925 |
|  | 47 | 171 | 336 | 552 | 775 | 1395 | 1916 |
|  | 47 | 170 | 306 | 526 | 967 | 1670 | 2050 |
|  | 45 | 170 | 323 | 487 | 800 | 1740 | 1790 |
|  | 47 | 162 | 356 | 553 | 791 | 1226 | 1830 |
|  | 46 | 181 | 386 | 507 | 866 | 1247 | 1950 |
|  | 47 | 158 | 312 | 435 | 675 | 1950 | 1784 |
|  | 46 | 165 | 348 | 571 | 183 | 1833 | 2040 |
|  | 46 | 155 | 305 | 512 | 878 | 1543 | 1861 |
|  | 48 | 195 | 335 | 544 | 992 | 1581 | 1727 |
|  | 46 | 175 | 382 | 462 | 997 | 1734 | 2088 |
|  | 47 | 156 | 367 | 431 | 850 | 1834 | 2327 |
|  | 46 | 173 | 383 | 544 | 845 | 1693 | 2188 |
|  | 46 | 157 | 362 | 520 | 926 | 1733 | 1909 |
|  | 47 | 165 | 335 | 515 | 977 | 1638 | 1777 |
|  | 47 | 162 | 336 | 525 | 978 | 1588 | 1777 |
|  | 47 | 190 | 317 | 469 | 910 | 1544 | 1831 |
|  | 42 | 162 | 341 | 557 | 885 | 1740 | 1754 |
|  | 47 | 160 | 361 | 500 | 800 | 1555 | 1606 |
|  | 46 | 196 | 314 | 600 | 751 | 1633 | 2030 |
|  | 47 | 171 | 375 | 477 | 799 | 1933 | 1622 |
|  | 45 | 162 | 321 | 477 | 912 | 1354 | 2026 |
|  | 47 | 168 | 351 | 562 | 777 | 1452 | 1685 |
|  | 46 | 163 | 306 | 500 | 900 | 1663 | 1774 |
|  | 45 | 175 | 324 | 663 | 924 | 1638 | 2033 |
|  | 45 | 150 | 384 | 469 | 776 | 1490 | 1854 |
|  | 48 | 175 | 350 | 500 | 826 | 1452 | 1656 |
|  | 48 | 183 | 371 | 562 | 966 | 1474 | 1694 |
|  | 47 | 161 | 356 | 497 | 1000 | 1733 | 1620 |
|  | 47 | 171 | 360 | 485 | 1000 | 1444 | 2240 |
|  | 46 | 170 | 346 | 574 | 990 | 1590 | 1690 |
|  | 45 | 160 | 303 | 483 | 877 | 1640 | 1811 |
|  | 46 | 162 | 375 | 535 | 834 | 1399 | 1831 |
|  | 48 | 184 | 315 | 588 | 855 | 1766 | 1567 |
|  | 47 | 160 | 353 | 585 | 756 | 1499 | 1851 |
|  | 48 | 154 | 317 | 485 | 758 | 1890 | 1840 |
|  | 49 | 151 | 371 | 461 | 871 | 1770 | 1836 |
|  | 48 | 169 | 345 | 472 | 876 | 1626 | 1694 |
|  | 47 | 170 | 346 | 525 | 860 | 1513 | 1890 |
|  | 47 | 181 | 317 | 600 | 781 | 1734 | 1494 |
|  | 48 | 170 | 333 | 432 | 799 | 1490 | 1506 |
|  | 48 | 162 | 335 | 527 | 800 | 1493 | 1691 |
|  | 49 | 155 | 327 | 558 | 800 | 1594 | 1592 |
|  | 48 | 171 | 392 | 544 | 844 | 1699 | 1910 |
|  | 48 | 187 | 319 | 600 | 871 | 1890 | 1910 |
|  | 49 | 186 | 367 | 505 | 855 | 1890 | 1910 |
| Group 2 Phytezyme | 48 | 134 | 350 | 420 | 710 | 1440 | 1802 |
|  | 47 | 151 | 321 | 473 | 755 | 1370 | 1770 |
|  | 48 | 148 | 351 | 570 | 820 | 1318 | 1620 |
|  | 48 | 156 | 336 | 513 | 711 | 1383 | 1561 |
|  | 47 | 168 | 340 | 520 | 800 | 1440 | 1440 |
|  | 46 | 152 | 296 | 490 | 710 | 1520 | 1361 |
|  | 45 | 139 | 321 | 440 | 726 | 1603 | 1385 |
|  | 46 | 164 | 350 | 450 | 834 | 1801 | 1561 |
|  | 47 | 152 | 335 | 456 | 600 | 1211 | 1603 |
|  | 48 | 160 | 327 | 420 | 766 | 1126 | 1460 |
|  | 47 | 171 | 321 | 524 | 777 | 1233 | 1586 |
|  | 45 | 147 | 331 | 403 | 800 | 1181 | 1646 |
|  | 48 | 154 | 347 | 420 | 831 | 1230 | 1550 |
|  | 47 | 167 | 350 | 460 | 900 | 1255 | 1570 |
|  | 46 | 144 | 360 | 426 | 824 | 1440 | 1611 |
|  | 45 | 165 | 320 | 505 | 713 | 1408 | 1616 |
|  | 46 | 170 | 322 | 444 | 730 | 1513 | 1621 |
|  | 48 | 162 | 335 | 520 | 806 | 1418 | 1520 |
|  | 46 | 160 | 296 | 576 | 731 | 1314 | 1684 |
|  | 49 | 110 | 340 | 513 | 829 | 1446 | 1760 |
|  | 48 | 170 | 330 | 407 | 822 | 1504 | 1490 |
|  | 48 | 148 | 300 | 460 | 807 | 1441 | 1866 |
|  | 49 | 166 | 350 | 483 | 719 | 1333 | 1626 |
|  | 47 | 141 | 351 | 426 | 806 | 1000 | 1953 |
|  | 46 | 173 | 321 | 488 | 740 | 1110 | 1650 |
|  | 45 | 157 | 316 | 500 | 699 | 1130 | 1561 |
|  | 48 | 163 | 350 | 503 | 751 | 1154 | 1440 |
|  | 47 | 149 | 344 | 500 | 809 | 1431 | 1690 |
|  | 46 | 155 | 344 | 467 | 811 | 1421 | 1693 |
|  | 48 | 160 | 335 | 420 | 750 | 1440 | 1390 |
|  | 48 | 151 | 344 | 487 | 726 | 1370 | 1611 |
|  | 48 | 144 | 327 | 424 | 788 | 1333 | 1299 |
|  | 49 | 168 | 300 | 413 | 676 | 1484 | 1490 |
|  | 47 | 169 | 350 | 477 | 640 | 1535 | 1747 |
|  | 46 | 154 | 321 | 445 | 688 | 1640 | 1744 |
|  | 45 | 147 | 352 | 430 | 817 | 1555 | 1625 |
|  | 47 | 176 | 350 | 417 | 766 | 1494 | 1499 |
|  | 48 | 166 | 336 | 503 | 721 | 1538 | 1591 |
|  | 49 | 132 | 305 | 426 | 710 | 1439 | 1651 |
|  | 48 | 160 | 365 | 429 | 818 | 1454 | 1611 |
|  | 47 | 163 | 353 | 510 | 711 | 1288 | 1566 |
|  | 47 | 144 | 347 | 530 | 806 | 1390 | 1563 |
|  | 48 | 151 | 348 | 444 | 801 | 1550 | 1900 |
|  | 48 | 153 | 350 | 455 | 719 | 1634 | 1351 |
|  | 47 | 142 | 360 | 490 | 703 | 1480 | 1380 |
|  | 49 | 156 | 265 | 436 | 609 | 1131 | 1351 |
|  | 48 | 149 | 320 | 469 | 721 | 1199 | 1380 |
|  | 48 | 156 | 327 | 423 | 711 | 1494 | 1380 |
|  | 47 | 149 | 265 | 408 | 790 | 1530 | 1351 |
|  | 46 | 144 | 265 | 413 | 701 | 1641 | 1351 |
| Group 3 Control | 46 | 158 | 315 | 472 | 657 | 1131 | 1460 |
|  | 48 | 141 | 342 | 503 | 719 | 1440 | 1330 |
|  | 47 | 156 | 335 | 430 | 688 | 1370 | 1470 |
|  | 46 | 140 | 300 | 447 | 716 | 1320 | 1646 |
|  | 49 | 159 | 340 | 476 | 801 | 1419 | 1561 |
|  | 47 | 172 | 316 | 490 | 690 | 1140 | 1344 |
|  | 48 | 163 | 320 | 406 | 711 | 1193 | 1590 |
|  | 46 | 147 | 320 | 485 | 830 | 1000 | 1563 |
|  | 47 | 148 | 323 | 489 | 624 | 1240 | 1484 |
|  | 47 | 152 | 302 | 446 | 666 | 1253 | 1990 |
|  | 48 | 152 | 323 | 423 | 714 | 1191 | 1360 |
|  | 49 | 153 | 360 | 416 | 800 | 1113 | 1440 |
|  | 47 | 154 | 309 | 409 | 820 | 1288 | 1480 |
|  | 46 | 141 | 206 | 430 | 713 | 1441 | 1466 |
|  | 48 | 136 | 205 | 450 | 641 | 1533 | 1560 |
|  | 48 | 141 | 305 | 467 | 688 | 1641 | 1290 |
|  | 48 | 158 | 300 | 486 | 699 | 1341 | 1691 |
|  | 49 | 157 | 360 | 441 | 720 | 1470 | 1387 |
|  | 47 | 163 | 320 | 452 | 844 | 1191 | 1490 |
|  | 46 | 126 | 309 | 443 | 823 | 1220 | 1566 |
|  | 46 | 166 | 206 | 436 | 771 | 1101 | 1560 |
|  | 46 | 165 | 305 | 444 | 733 | 1103 | 1433 |
|  | 48 | 149 | 305 | 423 | 900 | 1280 | 1581 |
|  | 47 | 149 | 300 | 434 | 822 | 1000 | 1391 |
|  | 49 | 144 | 360 | 432 | 900 | 1903 | 1599 |
|  | 46 | 171 | 320 | 454 | 733 | 1370 | 2000 |
|  | 47 | 163 | 309 | 461 | 650 | 1440 | 1910 |
|  | 48 | 152 | 312 | 485 | 688 | 1595 | 1866 |
|  | 47 | 154 | 305 | 476 | 657 | 1611 | 1984 |
|  | 46 | 148 | 327 | 426 | 612 | 1221 | 1761 |
|  | 48 | 153 | 357 | 416 | 717 | 1334 | 1687 |
|  | 47 | 142 | 302 | 407 | 829 | 1341 | 1770 |
|  | 49 | 154 | 300 | 484 | 801 | 1374 | 1860 |
|  | 46 | 162 | 309 | 419 | 900 | 1119 | 1706 |
|  | 48 | 170 | 303 | 450 | 711 | 1981 | 1440 |
|  | 47 | 120 | 218 | 426 | 718 | 1333 | 1390 |
|  | 46 | 146 | 310 | 440 | 733 | 1454 | 1560 |
|  | 49 | 142 | 347 | 453 | 813 | 1455 | 1406 |
|  | 48 | 158 | 344 | 456 | 712 | 1611 | 1390 |
|  | 48 | 154 | 320 | 406 | 709 | 1366 | 1399 |
|  | 47 | 148 | 365 | 454 | 620 | 1441 | 1466 |
|  | 47 | 155 | 220 | 443 | 633 | 1199 | 1420 |
|  | 48 | 150 | 235 | 444 | 688 | 1191 | 1511 |
|  | 46 | 140 | 316 | 490 | 804 | 1341 | 1399 |
|  | 47 | 160 | 324 | 487 | 726 | 1931 | 1311 |
|  | 49 | 143 | 365 | 476 | 699 | 1611 | 1388 |
|  | 46 | 150 | 220 | 407 | 681 | 1733 | 1680 |
|  | 48 | 156 | 223 | 487 | 800 | 1891 | 1575 |
|  | 47 | 144 | 235 | 430 | 804 | 1833 | 1390 |
|  | 47 | 141 | 319 | 450 | 804 | 1930 | 1390 |

**Statistical analysis of Supplementation of immobilized Carlsberg Subtilisin in poultry feed.**

| **Descriptive** | | | | | | | | |
| --- | --- | --- | --- | --- | --- | --- | --- | --- |
|  | | N | Mean | Std. Deviation | Std. Error | 95% Confidence Interval for Mean | |  |
|  |  |  |  |  |  | Lower Bound | Upper Bound |  |
| Day4 | Protease | 50 | 46,8600 | 1,08816 | ,15389 | 46,5507 | 47,1693 |  |
|  | Phytezyme | 49 | 47,2041 | 1,15433 | ,16490 | 46,8725 | 47,5356 |  |
|  | Control | 50 | 47,3000 | 1,01519 | ,14357 | 47,0115 | 47,5885 |  |
|  | Total | 149 | 47,1208 | 1,09614 | ,08980 | 46,9434 | 47,2983 |  |
| Day10 | Protease | 50 | 168,0600 | 11,37920 | 1,60926 | 164,8261 | 171,2939 |  |
|  | Phytezyme | 49 | 155,2245 | 11,02887 | 1,57555 | 152,0566 | 158,3924 |  |
|  | Control | 50 | 151,6400 | 9,80995 | 1,38734 | 148,8520 | 154,4280 |  |
|  | Total | 149 | 158,3289 | 12,82494 | 1,05066 | 156,2526 | 160,4051 |  |
| Day16 | Protease | 50 | 343,9200 | 25,58048 | 3,61763 | 336,6501 | 351,1899 |  |
|  | Phytezyme | 48 | 333,5417 | 20,21687 | 2,91805 | 327,6713 | 339,4120 |  |
|  | Control | 50 | 318,4200 | 22,20598 | 3,14040 | 312,1091 | 324,7309 |  |
|  | Total | 148 | 331,9392 | 24,99993 | 2,05498 | 327,8781 | 336,0003 |  |
| Day22 | Protease | 50 | 522,8800 | 48,90793 | 6,91663 | 508,9805 | 536,7795 |  |
|  | Phytezyme | 48 | 466,7708 | 43,11945 | 6,22376 | 454,2503 | 479,2914 |  |
|  | Control | 50 | 449,1400 | 26,96257 | 3,81308 | 441,4773 | 456,8027 |  |
|  | Total | 148 | 479,7703 | 51,38624 | 4,22392 | 471,4228 | 488,1177 |  |
| Day28 | Protease | 50 | 863,7800 | 79,10811 | 11,18758 | 841,2977 | 886,2623 |  |
|  | Phytezyme | 48 | 754,5417 | 62,08640 | 8,96140 | 736,5137 | 772,5697 |  |
|  | Control | 48 | 735,9167 | 75,58082 | 10,90915 | 713,9703 | 757,8631 |  |
|  | Total | 146 | 785,8288 | 91,97784 | 7,61214 | 770,7837 | 800,8739 |  |
| Day34 | Protease | 49 | 1280,1837 | 136,15030 | 19,45004 | 1241,0767 | 1319,2906 |  |
|  | Phytezyme | 48 | 1113,4375 | 124,27370 | 17,93736 | 1077,3522 | 1149,5228 |  |
|  | Control | 48 | 1072,7292 | 112,98460 | 16,30792 | 1039,9219 | 1105,5365 |  |
|  | Total | 145 | 1156,3103 | 153,45668 | 12,74388 | 1131,1211 | 1181,4996 |  |
| Day40 | Protease | 49 | 1613,2857 | 163,82397 | 23,40342 | 1566,2300 | 1660,3415 |  |
|  | Phytezyme | 48 | 1387,4583 | 143,02774 | 20,64428 | 1345,9274 | 1428,9893 |  |
|  | Control | 48 | 1351,5833 | 184,83711 | 26,67894 | 1297,9122 | 1405,2544 |  |
|  | Total | 145 | 1451,8966 | 200,93627 | 16,68685 | 1418,9137 | 1484,8794 |  |
| Day46 | Protease | 49 | 1821,7755 | 175,09120 | 25,01303 | 1771,4834 | 1872,0676 |  |
|  | Phytezyme | 45 | 1583,7556 | 128,64471 | 19,17722 | 1545,1064 | 1622,4047 |  |
|  | Control | 48 | 1554,3958 | 186,94677 | 26,98344 | 1500,1121 | 1608,6795 |  |
|  | Total | 142 | 1655,9648 | 204,89231 | 17,19418 | 1621,9731 | 1689,9565 |  |

| **Test of Homogeneity of Variances** | | | | | |
| --- | --- | --- | --- | --- | --- |
|  | | Levene Statistic | df1 | df2 | Sig. |
| Day4 | Based on Mean | ,347 | 2 | 146 | ,707 |
|  | Based on Median | ,487 | 2 | 146 | ,616 |
|  | Based on Median and with adjusted df | ,487 | 2 | 145,284 | ,616 |
|  | Based on trimmed mean | ,353 | 2 | 146 | ,703 |
| Day10 | Based on Mean | ,588 | 2 | 146 | ,557 |
|  | Based on Median | ,609 | 2 | 146 | ,545 |
|  | Based on Median and with adjusted df | ,609 | 2 | 143,716 | ,545 |
|  | Based on trimmed mean | ,598 | 2 | 146 | ,551 |
| Day16 | Based on Mean | 2,353 | 2 | 145 | ,099 |
|  | Based on Median | 2,344 | 2 | 145 | ,100 |
|  | Based on Median and with adjusted df | 2,344 | 2 | 142,398 | ,100 |
|  | Based on trimmed mean | 2,468 | 2 | 145 | ,088 |
| Day22 | Based on Mean | 8,080 | 2 | 145 | ,000 |
|  | Based on Median | 7,681 | 2 | 145 | ,001 |
|  | Based on Median and with adjusted df | 7,681 | 2 | 120,990 | ,001 |
|  | Based on trimmed mean | 7,994 | 2 | 145 | ,001 |
| Day28 | Based on Mean | 1,210 | 2 | 143 | ,301 |
|  | Based on Median | ,897 | 2 | 143 | ,410 |
|  | Based on Median and with adjusted df | ,897 | 2 | 130,696 | ,410 |
|  | Based on trimmed mean | 1,102 | 2 | 143 | ,335 |
| Day34 | Based on Mean | ,587 | 2 | 142 | ,557 |
|  | Based on Median | ,903 | 2 | 142 | ,408 |
|  | Based on Median and with adjusted df | ,903 | 2 | 138,336 | ,408 |
|  | Based on trimmed mean | ,628 | 2 | 142 | ,535 |
| Day40 | Based on Mean | 1,412 | 2 | 142 | ,247 |
|  | Based on Median | 1,512 | 2 | 142 | ,224 |
|  | Based on Median and with adjusted df | 1,512 | 2 | 140,204 | ,224 |
|  | Based on trimmed mean | 1,423 | 2 | 142 | ,244 |
| Day46 | Based on Mean | 2,755 | 2 | 139 | ,067 |
|  | Based on Median | 2,394 | 2 | 139 | ,095 |
|  | Based on Median and with adjusted df | 2,394 | 2 | 125,382 | ,095 |
|  | Based on trimmed mean | 2,709 | 2 | 139 | ,070 |

| **ANOVA** | | | | | | |
| --- | --- | --- | --- | --- | --- | --- |
|  | | Sum of Squares | df | Mean Square | F | Sig. |
| Day4 | Between Groups | 5,346 | 2 | 2,673 | 2,263 | ,108 |
|  | Within Groups | 172,479 | 146 | 1,181 |  |  |
|  | Total | 177,826 | 148 |  |  |  |
| Day10 | Between Groups | 7444,015 | 2 | 3722,008 | 32,157 | ,000 |
|  | Within Groups | 16898,871 | 146 | 115,746 |  |  |
|  | Total | 24342,886 | 148 |  |  |  |
| Day16 | Between Groups | 16438,676 | 2 | 8219,338 | 15,799 | ,000 |
|  | Within Groups | 75435,777 | 145 | 520,247 |  |  |
|  | Total | 91874,453 | 147 |  |  |  |
| Day22 | Between Groups | 147944,410 | 2 | 73972,205 | 44,651 | ,000 |
|  | Within Groups | 240215,779 | 145 | 1656,661 |  |  |
|  | Total | 388160,189 | 147 |  |  |  |
| Day28 | Between Groups | 470384,556 | 2 | 235192,278 | 44,470 | ,000 |
|  | Within Groups | 756304,163 | 143 | 5288,840 |  |  |
|  | Total | 1226688,719 | 145 |  |  |  |
| Day34 | Between Groups | 1175432,396 | 2 | 587716,198 | 37,667 | ,000 |
|  | Within Groups | 2215616,639 | 142 | 15602,934 |  |  |
|  | Total | 3391049,034 | 144 |  |  |  |
| Day40 | Between Groups | 1958597,865 | 2 | 979298,932 | 36,068 | ,000 |
|  | Within Groups | 3855457,583 | 142 | 27151,110 |  |  |
|  | Total | 5814055,448 | 144 |  |  |  |
| Day46 | Between Groups | 2076984,503 | 2 | 1038492,252 | 37,569 | ,000 |
|  | Within Groups | 3842316,321 | 139 | 27642,563 |  |  |
|  | Total | 5919300,824 | 141 |  |  |  |

| **ANOVA** | | | | | | |
| --- | --- | --- | --- | --- | --- | --- |
|  | | Sum of Squares | df | Mean Square | F | Sig. |
| Day4 | Between Groups | 5,346 | 2 | 2,673 | 2,263 | ,108 |
|  | Within Groups | 172,479 | 146 | 1,181 |  |  |
|  | Total | 177,826 | 148 |  |  |  |
| Day10 | Between Groups | 7444,015 | 2 | 3722,008 | 32,157 | ,000 |
|  | Within Groups | 16898,871 | 146 | 115,746 |  |  |
|  | Total | 24342,886 | 148 |  |  |  |
| Day16 | Between Groups | 16438,676 | 2 | 8219,338 | 15,799 | ,000 |
|  | Within Groups | 75435,777 | 145 | 520,247 |  |  |
|  | Total | 91874,453 | 147 |  |  |  |
| Day22 | Between Groups | 147944,410 | 2 | 73972,205 | 44,651 | ,000 |
|  | Within Groups | 240215,779 | 145 | 1656,661 |  |  |
|  | Total | 388160,189 | 147 |  |  |  |
| Day28 | Between Groups | 470384,556 | 2 | 235192,278 | 44,470 | ,000 |
|  | Within Groups | 756304,163 | 143 | 5288,840 |  |  |
|  | Total | 1226688,719 | 145 |  |  |  |
| Day34 | Between Groups | 1175432,396 | 2 | 587716,198 | 37,667 | ,000 |
|  | Within Groups | 2215616,639 | 142 | 15602,934 |  |  |
|  | Total | 3391049,034 | 144 |  |  |  |
| Day40 | Between Groups | 1958597,865 | 2 | 979298,932 | 36,068 | ,000 |
|  | Within Groups | 3855457,583 | 142 | 27151,110 |  |  |
|  | Total | 5814055,448 | 144 |  |  |  |
| Day46 | Between Groups | 2076984,503 | 2 | 1038492,252 | 37,569 | ,000 |
|  | Within Groups | 3842316,321 | 139 | 27642,563 |  |  |
|  | Total | 5919300,824 | 141 |  |  |  |

| **Day4** | | |
| --- | --- | --- |
| Duncan^a,b^ | | |
| Groups | N | Subset for alpha = 0.05 |
|  |  | 1 |
| Protease | 50 | 46,8600 |
| Phytezyme | 49 | 47,2041 |
| Control | 50 | 47,3000 |
| Sig. |  | ,057 |
| Means for groups in homogeneous subsets are displayed. | | |
| a. Uses Harmonic Mean Sample Size = 49,662. | | |
| b. The group sizes are unequal. The harmonic mean of the group sizes is used. Type I error levels are not guaranteed. | | |

| **Day10** | | | |
| --- | --- | --- | --- |
| Duncan^a,b^ | | | |
| Groups | N | Subset for alpha = 0.05 | |
|  |  | 1 | 2 |
| Control | 50 | 151,6400 |  |
| Phytezyme | 49 | 155,2245 |  |
| Protease | 50 |  | 168,0600 |
| Sig. |  | ,099 | 1,000 |
| Means for groups in homogeneous subsets are displayed. | | | |
| a. Uses Harmonic Mean Sample Size = 49,662. | | | |
| b. The group sizes are unequal. The harmonic mean of the group sizes is used. Type I error levels are not guaranteed. | | | |

| **Day16** | | | | |
| --- | --- | --- | --- | --- |
| Duncan^a,b^ | | | | |
| Groups | N | Subset for alpha = 0.05 | | |
|  |  | 1 | 2 | 3 |
| Control | 50 | 318,4200 |  |  |
| Phytezyme | 48 |  | 333,5417 |  |
| Protease | 50 |  |  | 343,9200 |
| Sig. |  | 1,000 | 1,000 | 1,000 |
| Means for groups in homogeneous subsets are displayed. | | | | |
| a. Uses Harmonic Mean Sample Size = 49,315. | | | | |
| b. The group sizes are unequal. The harmonic mean of the group sizes is used. Type I error levels are not guaranteed. | | | | |

| **Day22** | | | | |
| --- | --- | --- | --- | --- |
| Duncan^a,b^ | | | | |
| Groups | N | Subset for alpha = 0.05 | | |
|  |  | 1 | 2 | 3 |
| Control | 50 | 449,1400 |  |  |
| Phytezyme | 48 |  | 466,7708 |  |
| Protease | 50 |  |  | 522,8800 |
| Sig. |  | 1,000 | 1,000 | 1,000 |
| Means for groups in homogeneous subsets are displayed. | | | | |
| a. Uses Harmonic Mean Sample Size = 49,315. | | | | |
| b. The group sizes are unequal. The harmonic mean of the group sizes is used. Type I error levels are not guaranteed. | | | | |

| **Day28** | | | |
| --- | --- | --- | --- |
| Duncan^a,b^ | | | |
| Groups | N | Subset for alpha = 0.05 | |
|  |  | 1 | 2 |
| Control | 48 | 735,9167 |  |
| Phytezyme | 48 | 754,5417 |  |
| Protease | 50 |  | 863,7800 |
| Sig. |  | ,209 | 1,000 |
| Means for groups in homogeneous subsets are displayed. | | | |
| a. Uses Harmonic Mean Sample Size = 48,649. | | | |
| b. The group sizes are unequal. The harmonic mean of the group sizes is used. Type I error levels are not guaranteed. | | | |

| **Day34** | | | |
| --- | --- | --- | --- |
| Duncan^a,b^ | | | |
| Groups | N | Subset for alpha = 0.05 | |
|  |  | 1 | 2 |
| Control | 48 | 1072,7292 |  |
| Phytezyme | 48 | 1113,4375 |  |
| Protease | 49 |  | 1280,1837 |
| Sig. |  | ,111 | 1,000 |
| Means for groups in homogeneous subsets are displayed. | | | |
| a. Uses Harmonic Mean Sample Size = 48,329. | | | |
| b. The group sizes are unequal. The harmonic mean of the group sizes is used. Type I error levels are not guaranteed. | | | |

| **Day40** | | | |
| --- | --- | --- | --- |
| Duncan^a,b^ | | | |
| Groups | N | Subset for alpha = 0.05 | |
|  |  | 1 | 2 |
| Control | 48 | 1351,5833 |  |
| Phytezyme | 48 | 1387,4583 |  |
| Protease | 49 |  | 1613,2857 |
| Sig. |  | ,286 | 1,000 |
| Means for groups in homogeneous subsets are displayed. | | | |
| a. Uses Harmonic Mean Sample Size = 48,329. | | | |
| b. The group sizes are unequal. The harmonic mean of the group sizes is used. Type I error levels are not guaranteed. | | | |

| **Day46** | | | |
| --- | --- | --- | --- |
| Duncan^a,b^ | | | |
| Groups | N | Subset for alpha = 0.05 | |
|  |  | 1 | 2 |
| Control | 48 | 1554,3958 |  |
| Phytezyme | 45 | 1583,7556 |  |
| Protease | 49 |  | 1821,7755 |
| Sig. |  | ,392 | 1,000 |
| Means for groups in homogeneous subsets are displayed. | | | |
| a. Uses Harmonic Mean Sample Size = 47,271. | | | |
| b. The group sizes are unequal. The harmonic mean of the group sizes is used. Type I error levels are not guaranteed. | | | |
