# Supplementary material for: Immobilization and docking studies of Carlsberg subtilisin for application in poultry industry
Source: PLoS One. 2023 Aug 16;18(8):e0269717. doi: 10.1371/journal.pone.0269717 (PMC10431679; doi:10.1371/journal.pone.0269717)
Supplement: Attachment — Submitted filename: Response to reviewers.docx [file pone.0269717.s005.docx]

PONE-D-21-14945
Immobilization and docking studies of Thermophilic Carlsberg Subtilisin on Bentonite as a poultry feed supplement
PLOS ONE

Reviewer 2:

Thank you for careful evaluation of the Manuscript. I have tried my best to make all the suggested changes to the manuscript.

Reviewer #3: Manuscript number: PONE-D-21-14945
Title: Immobilization and docking studies of Thermophilic Carlsberg Subtilisin on Bentonite
as a poultry feed supplement

The manuscript entitled ‘Immobilization and docking studies of Thermophilic Carlsberg Subtilisin on Bentonite as a poultry feed supplement' is an interesting piece of work that gives details about immobilized thermophilic Carlsberg Subtilisin on bentonite and its application to poultry feed supplement'. They prepared the Carlsberg Subtilisin enzyme, and then immobilized on to bentonite surface by physical adsorption, and then feed to poultry feeding. The results are deserved to be published in enzyme technology area. However, the level of results and discussion is not considered enough to merit its publishing in PLOS ONE. Some of the suggested changes to be made are given below.

Major
Q1. The authors should describe advantage of bentonite relevant with enzyme immobilization. This discussion would be very important to address originality of the manuscript.

The discussion section includes all the necessary information highlighting the immobilization of subtilisin to bentonite and its impact on the poultry in terms of weight gain. As bentonite is an inexpensive matrix that does not have toxicity and chemical reactivity towards poultry making it most suitable solid support material for immobilization of Carlsberg subtilisin enzyme for potential use in poultry industry. Moreover, Bentonite is already an approved ingredient used in Asian poultry industry with an intention to control wet droppings making it most ideal solid support.

Q2. Physico-chemical characterization of bentonite should be discussed.

A separate comparative study is designed which addresses the Kinetics of immobilization in comparison with free enzyme. Additionally, the immobilization was confirmed by performing the physico-chemical evaluation through Raman, FTIR, SEM and XRD. The study includes the MALDI-TOF MS/MS Analysis of the Carlsberg subtilisin with O18 tagging of immobilized and free enzyme further digested with trypsin to evaluate the precise amino acid involved in binding of the protein to the bentonite. A comparative study design to evaluate the amino acid attachment pattern. The study also includes the comparative analysis of metal ions and inhibitors effect on immobilized and free enzyme.

Q3. The author conclude that bentonite is better than carbon materials for protease immobilization. However, depending on experimental condition, the result might be changed. This information must be clear in the manuscript.

The carbon-based adsorption diet formulation of proteases has a drawback of fungal contamination increasing the risks to birds. As the temperate and humid regions have greater chances of fungal growth. During experimentation upon storage for more than two weeks, we observed mold growing on the product requiring the need to add some preservatives to the formulations. Whereas this study is designed in a way that no foreign or unapproved ingredient added to feed might increase the risks to the birds.

Q4. Provide the supporting literature information about bentonite for enzyme immobilization (additional comparison Table) to justify the significance of this study.

The Bentonite is mostly used in feed as a wet liter controlling agent, making this easier to choose and found it to be a good immobilizing agent for subtilisin.

Q5. Was determined the full loading of immobilized enzyme prepared under the optimized immobilization conditions? This information must be clear in the manuscript.

The optimized conditions were observed to make the loading for immobilization and is addressed in the manuscript as well. I have added the free enzyme and immobilized enzyme comparison to highlight the impact of immobilized subtilisin to the bentonite but the results are already a part of another study as mentioned earlier and writing again will get plagiarized.

Q6. The loading capacity of enzyme immobilizaed prepared and the amount of attached enzyme: What were the optimum conditions? Please use numbers, no qualitative statements (high, good, higher, etc).

The suggested comments have been addressed in the reviewed document and suggestion changes have been made. The result and discussion section contains enzyme units in U/mg.

Q7. The kinetics and specific activity of immobilized protease on the bentonite based on the enzyme loading need to be more quantitative and should be given and discussed in the manuscript: Authors need to compare these results with other results in the literature.

The results of immobilization comparison and discussion is already a part of submitted paper as mentioned in Q4’s response. A full fledge study was designed stating the immobilization of enzyme to bentonite compared with free enzyme and discussed with respect to reported literature.

Q8. Comparison of the free and immobilized enzyme in terms of kinetic parameters: Authors need to compare these results with other results in the literature.

These parameters were studied and designed for another manuscript and shall be published with elaborated discussion of kinetic parameters and comparisons for the same study addressed in Q4.

Q9. The activity and stability of immobilized enzymes in bentonite carrier: The new biocatalysts presented were compared with a commercial material?? This information must be clear in the introduction.

The suggested information has been added to the introduction sections as well. The name of commercial enzyme supplement is given in methodology section referring to line number 229.

Q10. The influence of metal ions and some inhibitors on the enzyme activity were examined?

The effects were studied in another studied to be published in another paper as mentioned in Q4.

Q11. The effect of the detergents and organic solvents was studied?

The effect of detergent and organic solvents was tested in another study mentioned in Q4.

Q12. All discussion sections relevant with enzyme immobilization are very week, authors can improve it by suggesting the above citations such as how various in loading? why high activity? kinetic changes? And low reusability (may be leaching), what benefits of this system? etc.

The suggested changes have been made to the document. Majorly All of the concerning questions are related to another study that I have already designed for the second paper mentioned in Q4.

Q13. Bentonite is mineral materials which might affect weight gain to chicken. However, the author didn’t include any proper experimental condition to discuss with potential synergetic effect of them. This information must be clear in the manuscript.

The amount of bentonite was balanced out in the feed controls. A negative and positive controls were run in parallel to address the effect. Also, Bentonite is already being used in poultry feed to reduce the wet liter control. We added same amount of unabsorbed bentonite to evaluate the difference between both. We added same amount of bentonite to the commercial enzyme supplement test to reduce the false positive and negative results. The gist is highlighted in the manuscript line number 230-232.

*In compliance with data protection regulations, you may request that we remove your personal registration details at any time.* [*(Remove my information/details)*](https://www.editorialmanager.com/pone/login.asp?a=r)*. Please contact the publication office if you have any questions.*
